# Overview of the European post‐authorisation study register post‐authorization studies performed in Europe from September 2010 to December 2018

**DOI:** 10.1002/pds.5413

**Published:** 2022-02-11

**Authors:** Janet Sultana, Salvatore Crisafulli, Mariana Almas, Ippazio Cosimo Antonazzo, Esme Baan, Claudia Bartolini, Maria Paola Bertuccio, Fedele Bonifazi, Annalisa Capuano, Antonella Didio, Vera Ehrenstein, Mariagrazia Felisi, Carmen Ferrajolo, Andrea Fontana, Remy Francisca, Annie Fourrier‐Reglat, Joan Fortuny, Rosa Gini, Giulia Hyeraci, Christel Hoeve, Christos Kontogiorgis, Valentina Isgrò, Panagiotis‐Nikolaos Lalagkas, Luca L'Abbate, Deborah Layton, Annalisa Landi, Silvia Narduzzi, Leonardo Roque Pereira, Georgios Poulentzas, Concetta Rafaniello, Giuseppe Roberto, Giulia Scondotto, Liberata Sportiello, Maddalena Toma, Massoud Toussi, Katia Verhamme, Elisabetta Volpe, Gianluca Trifirò, On behalf the Working Group 3 of ENCePP (Inventory of EU data sources and methodological approaches for multisource studies)

**Affiliations:** ^1^ Pharmacy Department Mater Dei Hospital Msida Malta; ^2^ Exeter College of Medicine and Health Exeter UK; ^3^ Department of Medicine University of Verona Verona Italy; ^4^ Real World Solutions Department IQVIA Lisbon Portugal; ^5^ Research Centre on Public Health (CESP) University of Milan‐Bicocca Milan Italy; ^6^ Agenzia Regionale di Sanità della Toscana Florence Italy; ^7^ Department of Medical Informatics Erasmus Medical Centre Rotterdam The Netherlands; ^8^ TEDDY European Network of Excellence for Paediatric Clinical Research Pavia Italy; ^9^ Fondazione per la Ricerca Farmacologica Gianni Benzi Onlus Bari Italy; ^10^ Department of Experimental Medicine University of Campania "Vanvitelli" Naples Italy; ^11^ Campania Regional Center of Pharmacovigilance and Pharmacoepidemiology Naples Italy; ^12^ Department of Clinical Epidemiology Aarhus University Hospital Aarhus Denmark; ^13^ CVBF Consorzio per Valutazioni Biologiche e Farmacologiche Pavia Italy; ^14^ Unit of Biostatistics, Fondazione IRCCS Casa Sollievo della Sofferenza San Giovanni Rotondo Italy; ^15^ Univ. Bordeaux, INSERM, Bordeaux Population Health Research Center Team of Pharmacoepidemiology, UMR 1219 Bordeaux France; ^16^ RTI Health Solutions Barcelona Spain; ^17^ Democritus University of Thrace Alexandroupolis Greece; ^18^ Department of Diagnostics and Public Health University of Verona Verona Italy; ^19^ Data Science Hub Real World Solutions, IQVIA London UK; ^20^ Department of Datascience & Biostatistics University Medical Center Utrecht Utrecht The Netherlands

**Keywords:** EU PAS register, multi‐database studies, post‐authorization studies

## Abstract

**Background:**

The European post‐authorisation study (EU PAS) register is a repository launched in 2010 by the European Medicines Agency (EMA). All EMA‐requested PAS, commonly observational studies, must be recorded in this register. Multi‐database studies (MDS) leveraging secondary data have become an important strategy to conduct PAS in recent years, as reflected by the type of studies registered in the EU PAS register.

**Objectives:**

To analyse and describe PAS in the EU PAS register, with focus on MDS.

**Methods:**

Studies in the EU PAS register from inception to 31st December 2018 were described concerning transparency, regulatory obligations, scope, study type (e.g., observational study, clinical trial, survey, systematic review/meta‐analysis), study design, type of data collection and target population. MDS were defined as studies conducted through secondary use of >1 data source not linked at patient‐level. Data extraction was carried out independently by 14 centres with expertise in pharmacoepidemiology, using publicly available information in the EU PAS register including study protocol, whenever available, using a standardised data collection form. For validation purposes, a second revision of key fields for a 15% random sample of studies was carried out by a different centre. The inter‐rater reliability (IRR) was then calculated. Finally, to identify predictors of primary data collection‐based studies/versus those based on secondary use of healthcare databases) or MDS (vs. non‐MDS), odds ratios (OR) and 95% confidence intervals (CI) were calculated fitting univariate logistic regression models.

**Results:**

Overall, 1426 studies were identified. Clinical trials (*N* = 30; 2%), systematic reviews/meta‐analyses (*N* = 16; 1%) and miscellaneous study designs (*N* = 46; 3%) were much less common than observational studies (*N* = 1227; 86%). The protocol was available for 63% (*N* = 360) of 572 observational studies requested by a competent authority. Overall, 36% (*N* = 446) of observational studies were based fully or partially on primary data collection. Of 757 observational studies based on secondary use of data alone, 282 (37%) were MDS. Drug utilisation was significantly more common as a study scope in MDS compared to non‐MDS studies. The overall percentage agreement among collaborating centres that collected the data concerning study variables was highest for study type (93.5%) and lowest for type of secondary data (67.8%).

**Conclusions:**

Observational studies were the most common type of studies in the EU PAS register, but 30% used primary data, which is more resource‐intensive. Almost half of observational studies using secondary data were MDS. Data recording in the EU PAS register may be improved further, including more widespread availability of study protocols to improve transparency.

**Key Points**

- The present study provides a detailed description of all studies registered in the European post‐authorisation study (EU PAS) register from its inception till the end of 2018, focusing on various aspects of study design and multiple database studies specifically.
- Our results showed a steady increase in the number of observational studies registered, providing the most recent updated review of the EU PAS register.
- We leveraging a network of pharmacoepidemiologists from various centres belonging to European Network of Centres for Pharmacoepidemiology and Pharmacovigilance to collect additional and detailed information on methodological aspects of observational studies.
- The present study is the only one that evaluates the inter‐rater reliability of different investigators during the data collection process, thus providing indirect measure of the completeness and accuracy of the data collected

## 1 INTRODUCTION

The European post‐authorisation study (EU PAS) register is a publicly available repository of post‐authorisation (PAS) studies developed and supported by the European Medicines Agency (EMA) through the European Network of Centres for Pharmacoepidemiology and Pharmacovigilance (ENCePP).^1^ Current pharmacovigilance legislation in Europe requires to make the study protocols and summary of results publicly available for post‐authorisation safety studies (PASS) imposed as a condition to the Marketing Authorisation (RMP category 1) or as a specific obligation in the context of a Marketing Authorisation under exceptional circumstances (RMP category 2). This register is also aimed to host non‐imposed studies, such as those required as per Risk Management Plan (RMP category 3)^2^ and all observational studies performed on authorised medicinal products, including effectiveness studies. The EU PAS register is the platform through which such studies, as well as other non‐imposed studies, are made accessible online. Indeed, the aim of the EU PAS register, in line with ENCePP's mandate, is to improve the transparency of studies conducted within the EU area and beyond.

The EU PAS register has been described in detail in several previous studies. Engel & Almas et al, in a review of 189 PASS identified in the Pharmacovigilance Risk Assessment Committee (PRAC) Meeting Minutes from July 2012 to July 2015,^3^ searched the availability of those studies in the EU PAS and showed that only 93 of the studies were registered in the EU PAS register and among them only 43% had available protocols. Analysis of PRAC comments identified several methodological concerns, and limited reasoning behind the methodological decisions and feasibility considerations. Slightly more PASS used primary data collection (i.e., data prospectively collected for the purpose of the study) except those studies assessing drug utilisation where secondary use of already existing healthcare data was leveraged. Another review, conducted by Carroll et al and focusing on studies available on the EU PAS register as of October 2016,^4^ corroborated that primary data collection was more common in studies aimed at assessing safety and effectiveness and less common when assessing drug utilisation. In 2018, Vora et al and Farcas et al,^5^, ^6^ used the EU PAS register to explore specifically studies evaluating the implementation and impact of risk minimisation measures.

All these previously conducted EU PAS register‐based studies relied primary on the data reported within the register, without validation of the collected information or additional information on methodological aspects based on expert review. Another gap in these previous studies is the lack of focus on multiple‐database studies (MDS), which are observational studies conducted using more than one source of routinely collected data (e.g., claims database, electronic health records [EHRs]). MDS are of particular importance because they allow the accrual of a very large cohort of patients, which is of particular relevance to paediatric populations and rare diseases, as well as several other populations of special interest. Indeed, the number of MDS has been increasing over the years. Since EU PAS register is a platform for the mandatory recording of data on observational studies as per EU legislation, identifying and describing such studies and their impact on the landscape of observational research is therefore of great value as this has never been done to our knowledge.^7^

Given how quickly observational research is growing, the aim of the present descriptive study was to conduct an updated and detailed review of the studies that were registered in the EU PAS register from its inception till the end of 2018, providing additional information on data type (e.g., distinguishing between claims data, EHRs, etc.) and study design (e.g., distinguishing between descriptive studies, cohort studies, case–control studies, etc.). Another aim of this study was to conduct an assessment of the inter‐rater reliability following the collection of data.

## 2 METHODS

### 2.1 Data collection

**FIGURE 1:**
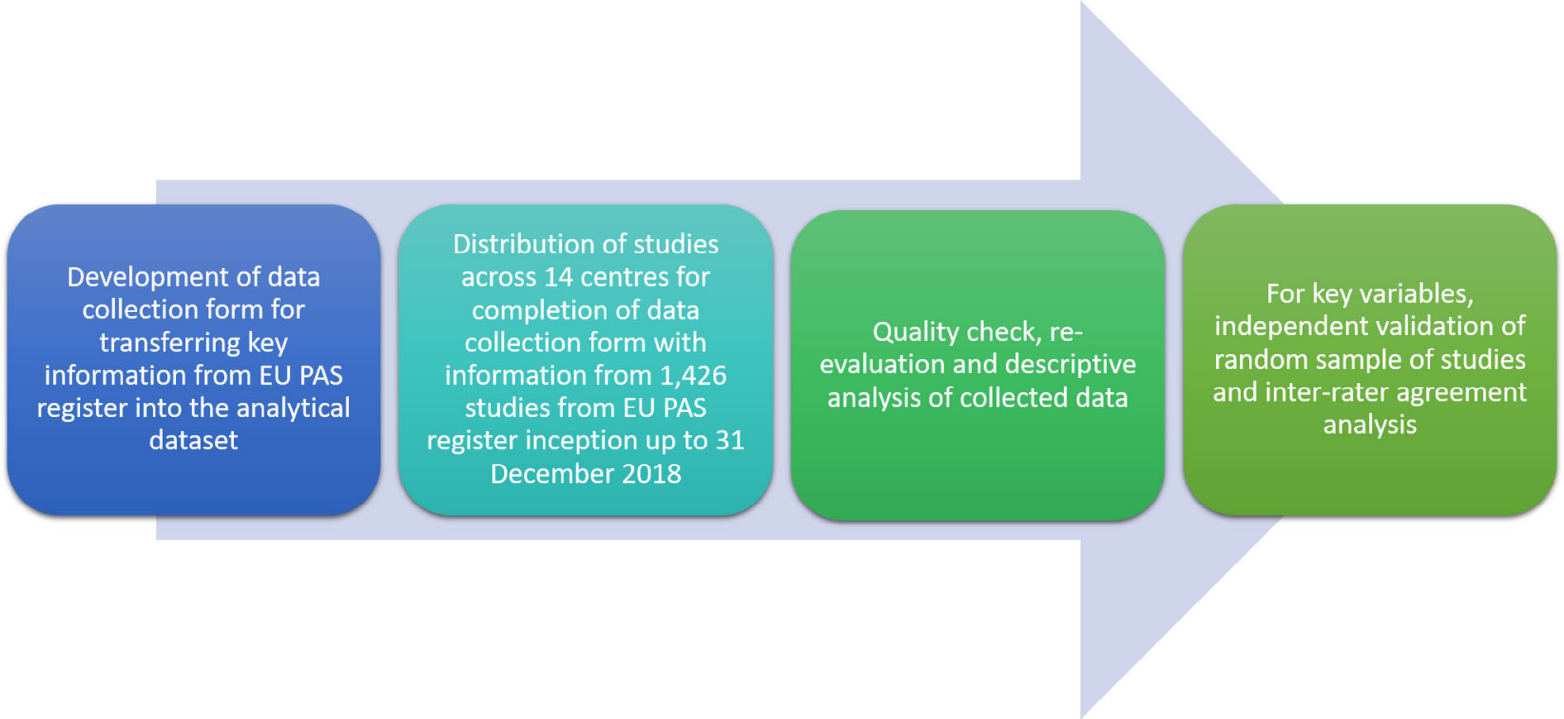
The methodological approach for EU PAS register review. EU PASs, European post‐authorization studies

A dataset containing all studies found in the EU PAS register from its inception to 31st December 2018 was provided by the EMA. The EU PAS register is publicly accessible online (http://www.encepp.eu/encepp/studySearch.htm). The data collection was carried out independently by 34 investigators from 13 academic centres or contract research organisations being part of ENCePP on common and detailed instructions and using the same electronic case report form (Figure 1). The resulting dataset contained data from the EU PAS register concerning different aspects of study transparency (ENCePP Seal, protocol and availability of publication), regulatory obligations, methodology, target population, scope and drug under study (chemically synthetised vs. biological drugs, orphan drugs). EU PAS register data was supplemented by retrieving data from study protocols or publications, if available, on source of funding, whether a study was an MDS (defined as a study using more than 1 source of already existing databases which could not be linked at patient level), study design, use of reference drug for formal comparison (if any). The full protocol for data collection, including the fields provided by the EMA, is provided in Appendix S1A. Once all the data was collected it was harmonised based on pre‐defined criteria.

To evaluate how consistent data collection was, data retrieved by investigators from a centre was checked by investigators from another centre for a 15% random sample of studies using the same protocol used for the main data collection; each investigator validated five studies. Any disagreements were resolved through the intervention of a third centre. This was done for the following key variables: setting, study type (new classification), study design, type of secondary data used, whether the study was an MDS, use of reference drug for formal comparison, drug type, whether the study drug was an orphan drug. After data collection was completed, automated quality check of data entry was conducted for the following fields: study type, data collection method, type of secondary data if applicable, whether the study was MDS, study type and study design. In brief, all investigators were asked to collect de novo data concerning these fields while blinded to previous assessment done by investigators belonging to a different centre.

### 2.2 Statistical analysis

The cumulative frequency of study registration in the EU PAS register was plotted. This was stratified by type of study, data collection and by MDS or non‐MDS status specifically. An overview of all studies was provided using descriptive statistics. This was done by stratifying at a high level by type of study (classified as clinical trials, observational studies, systematic review/meta‐analysis, questionnaire‐based surveys and others).

Finally, odds ratios (OR) and 95% confidence intervals (CIs) were calculated fitting univariate logistic regression models to investigate whether study‐related variables (e.g., study type, data source, etc.) were associated with the use of primary data versus use of secondary data as a (reference) and whether they were associated with non‐MDS versus MDS (reference) as a reference.

To better understand the data in the EU PAS register and add value to that data with the inclusion of further information related to methodology a large number of investigators was involved for collecting data.

Cohen's Kappa statistic was calculated in order to evaluate the inter‐rater reliability (IRR). This was done by single variables and macro‐categories consisting of several variables. Kappa estimates and their 95% CI were obtained by the resampling bootstrap method to account for heterogeneity between academic centres. Bootstrap replicate number was set equal to 100 000.

All statistical analyses were performed using R Studio (version1.3).

## 3 RESULTS

**FIGURE 2:**
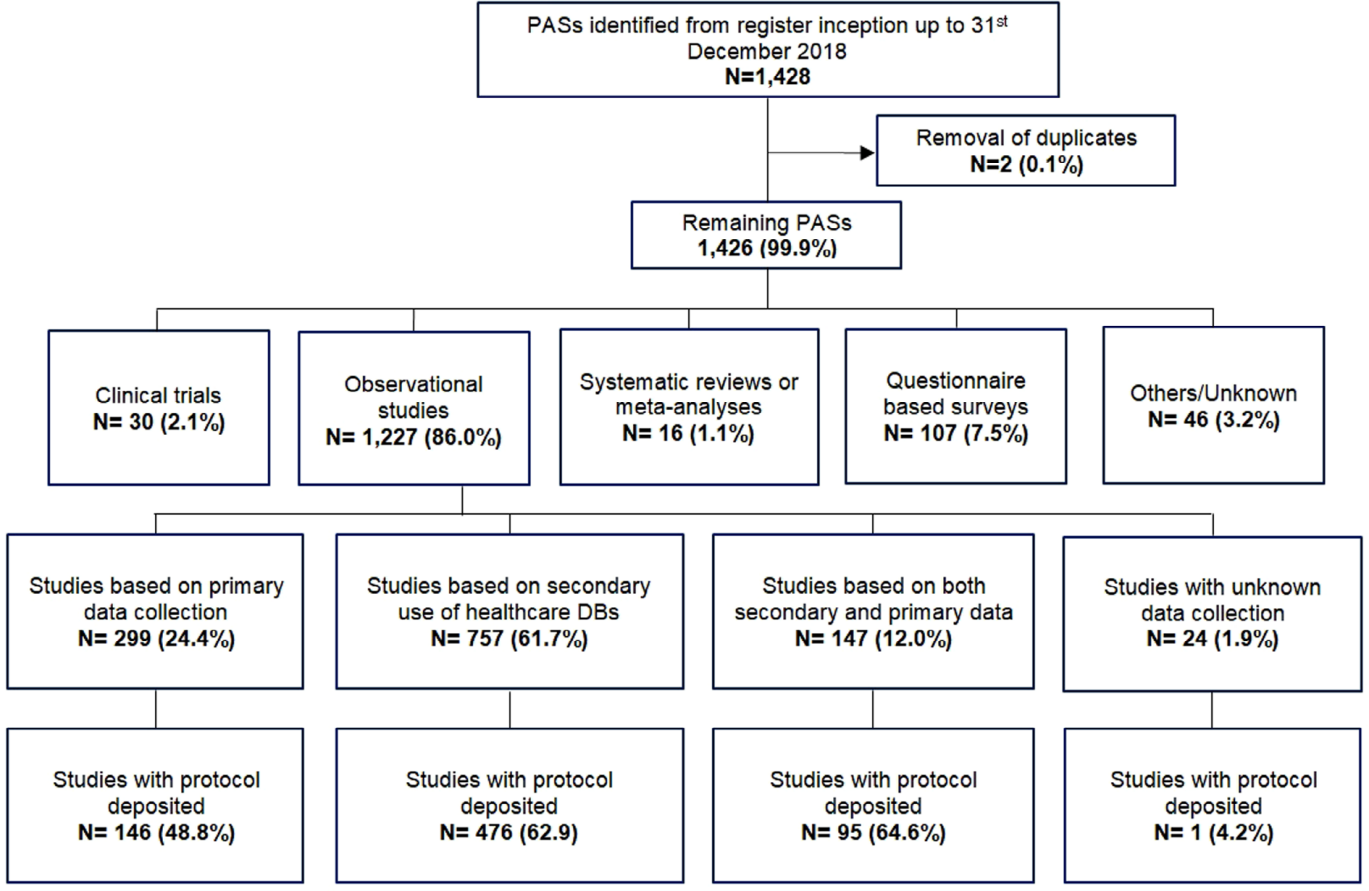
Flowchart of study types in the EU PAS register. ‘Others’ refers to any study design other than clinical trials, observational studies, systematic reviews, observational studies or questionnaires. Unknown refers to any study design that was not specified. EU PASs, European post‐authorization studies

After excluding two duplicates, a total of 1426 PASs were identified in the EU PAS register from its inception to 31st December 2018. The majority of studies were observational (*N* = 1227; 86.0%). Almost a quarter of all observational studies were based on primary data collection (*N* = 299; 24.4%; Figure 2). Two‐thirds of observational studies were based only on secondary use of existing healthcare data, that is, data not collected primarily for research purposes (*N* = 757; 61.7%). *N* = 299 (24.4%) were based on primary data and *N* = 147 (12.0%) were based on both primary data collection and secondary use of databases.

**FIGURE 3:**
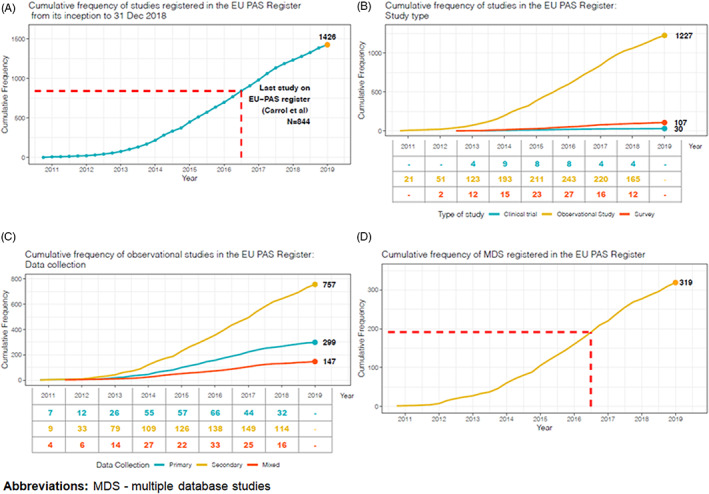
Cumulative frequency of studies recorded in the EU PAS register over time. The study by Carrol et al, is included as a benchmark of the most recent overview of the EU PAS register, including however only studies requested by the European Medicines Agency. MDS, multiple database studies; EU PASs, European post‐authorization studies

The frequency of studies registered in the EU PAS register showed a steady increase in the number of observational studies from 2010 to 2016, while a slight decrease was observed during the last 2 years under study (Figure 3). Almost a third of observational studies (*N* = 718; 58.5%) had a protocol deposited in the EU PAS register (Table 1), similar to most study types except for clinical trials, where only *N* = 11 (36.7%) had a protocol deposited. The protocol was available for 63% (*N* = 360) of observational studies requested by a competent authority. Only 6.9% of all observational studies had information on ENCePP Seal, while the availability of a publication was much more common: about a third of all observational studies had a publication available. Just under half of all studies were required by a competent authority. The most common type of risk management plan (RMP) classification for observational studies was RMP 3, that is, required by a competent authority (29.3%). Information on the RMP status was missing in 53 (4.3%) observational studies and it was classified as ‘non‐EU RMP only’ for 102 (8.3%) of them.

**TABLE 1:** Description of all studies identified in the EU PAS register

	Clinical trials *N* = 30 (%)	Observational studies *N* = 1227 (%)	Systematic reviews/meta‐analyses *N* = 16 (%)	Questionnaire‐based surveys *N* = 107 (%)
	*N* (%)	*N* (%)	*N* (%)	*N* (%)
Protocol deposited				
Yes	11 (36.7)	718 (58.5)	10 (62.5)	63 (58.9)
No	19 (63.3)	509 (41.5)	6 (37.5)	44 (41.1)
ENCePP Seal				
Yes	1 (3.3)	85 (6.9)	0 (0.0)	1 (0.9)
No	29 (96.7)	1142 (93.1)	16 (100.0)	106 (99.1)
Requested by a regulator				
Yes	10 (33.3)	571 (46.5)	5 (31.2)	68 (63.6)
No	18 (60.0)	637 (51.9)	11 (68.8)	39 (36.4)
Unknown	2 (6.7)	19 (1.5)	0 (0.0)	0 (0.0)
Status				
Planned	4 (13.3)	168 (13.7)	4 (25.0)	15 (14.0)
Ongoing	8 (26.7)	523 (42.6)	2 (12.5)	31 (29.0)
Finalised	18 (60.0)	536 (43.7)	10 (62.5)	61 (57.0)
Source of funding				
Pharmaceutical company	20 (66.7)	1005 (81.9)	10 (62.5)	97 (90.7)
National/international drug agency	0 (0)	53 (4.3)	3 (18.8)	1 (0.9)
Public entities excluding drug agencies	8 (26.7)	65 (5.3)	2 (12.5)	5 (4.7)
Self‐funded	1 (3.3)	23 (1.9)	1 (6.2)	0 (0)
More than one source	0 (0)	62 (5.1)	0 (0)	4 (3.7)
Unknown	1 (3.3)	19 (1.5)	0 (0)	0 (0)
RMP status				
Not applicable	15 (50.1)	600 (48.9)	11 (68.8)	25 (23.4)
EU RMP 1	1 (3.3)	83 (6.8)	0 (0)	10 (9.3)
EU RMP 2	1 (3.3)	30 (2.4)	0 (0)	3 (2.8)
EU RMP 3	6 (20.0)	359 (29.3)	3 (18.7)	42 (39.3)
Non‐EU RMP only	6 (20.0)	102 (8.3)	2 (12.5)	21 (19.6)
Missing—no info at all	1 (3.3)	53 (4.3)	0 (0)	6 (5.6)
Multidatabase study				
Yes	‐	319 (26.0)	‐	‐
No	‐	864 (70.4)	‐	‐
Unknown	‐	44 (3.6)	‐	‐
Data strategy				
Local data extraction and analysis, common protocol	‐	49 (4.0)	‐	‐
Local data extraction and central analysis on patient‐level raw data	‐	48 (3.9)	‐	‐
Study‐specific local data extraction in a common data model and central analysis	‐	40 (3.3)	‐	‐
General local data extraction in a common data model and central analysis	‐	48 (3.9)	‐	‐
Not applicable	‐	678 (55.3)	‐	‐
Unknown	‐	169 (13.8)	‐	‐
Missing data	‐	195 (15.8)	‐	‐
Product lifecylcle				
Pre‐marketing (for any indication)	4 (13.3)	4 (0.3)	0 (0.0)	0 (0.0)
Post‐marketing	17 (56.7)	1125 (91.7)	11 (68.8)	95 (88.8)
Not applicable	6 (20.0)	82 (6.7)	5 (31.2)	11 (10.3)
Unknown	3 (10.0)	16 (1.3)	0 (0.0)	1 (0.9)
Use of reference drug for formal comparison				
Yes	7 (23.3)	336 (27.4)	4 (25.0)	4 (25.0)
No	22 (73.4)	844 (68.8)	10 (62.5)	10 (62.5)
Unknown	1 (3.3)	47 (3.8)	2 (12.5)	2 (12.5)
Scope of the study				
Disease epidemiology	4 (13.3)	212 (17.3)	4 (9.5)	2 (12.5)
Risk assessment	13 (43.3)	696 (56.7)	15 (35.7)	11 (68.8)
Drug utilisation	4 (13.3)	444 (36.2)	6 (14.3)	1 (6.3)
Effectiveness evaluation	17 (56.7)	372 (30.3)	27 (64.3)	15 (93.8)
Other*	14 (46.7)	246 (20.0)	21 (50.0)	3 (18.8)
Population of interest—age				
Children	5 (16.7)	442 (36.0)	7 (43.8)	25 (23.4)
Adults	27 (90.0)	1103 (89.9)	15 (93.8)	105 (98.1)
Elderly persons	19 (63.3)	1008 (82.2)	13 (81.3)	98 (91.6)
Unknown	0 (0.0)	11 (0.9)	1 (6.3)	1 (0.9)
Population of interest—special populations				
Immunocompromised	1 (3.3)	87 (7.1)	1 (6.3)	4 (3.7)
Hepatic impairment	1 (3.3)	94 (7.7)	0 (0.0)	2 (1.9)
Renal impairment	2 (6.7)	108 (8.8)	0 (0.0)	5 (4.7)
Pregnant women	2 (6.7)	132 (10.8)	2 (12.5)	3 (2.8)
Breast‐feeding women	0 (0.0)	13 (1.1)	0 (0.0)	0 (0.0)
Other	3 (10.0)	73 (5.9)	2 (4.8)	1 (6.3)
Drug of interest—general				
Non‐biologic	19 (63.3)	754 (61.5)	10 (62.5)	70 (65.4)
Biologic	6 (20.0)	298 (24.3)	2 (12.5)	26 (24.3)
Both biologic and non‐biologic	0 (0.0)	25 (2.0)	0 (0.0)	1 (0.9)
None	5 (16.7)	109 (8.9)	4 (25.0)	8 (7.5)
Unknown	0 (0.0)	41 (3.3)	0 (0.0)	2 (1.9)
Drug of interest—orphan drugs				
Yes	5 (16.7)	157 (12.8)	0 (0.0)	14 (13.1)
No	25 (83.3)	1024 (83.5)	16 (100.0)	89 (83.2)
Unknown	0 (0.0)	46 (3.7)	0 (0.0)	4 (3.7)
Publication available				
Yes	12 (40.0)	359 (29.3)	7 (43.8)	29 (27.1)
No	18 (60.0)	868 (70.7)	9 (56.2)	78 (72.9)

Abbreviations: PASs, European post‐authorization studies; RMP, risk management plan.

The scope of most observational studies was risk assessment (56.7%), followed by drug utilisation (36.2%). A large proportion of observational studies was conducted included the elderly (82.2%). Only 36.0% (*N* = 442) of observational studies were conducted included children, and not all of these studies included children exclusively. With regards to drugs of interest, biologic drugs were studied in 24.3% of all observational studies; orphan drugs were the focus of 12.8% of observational studies.

**FIGURE 4:**
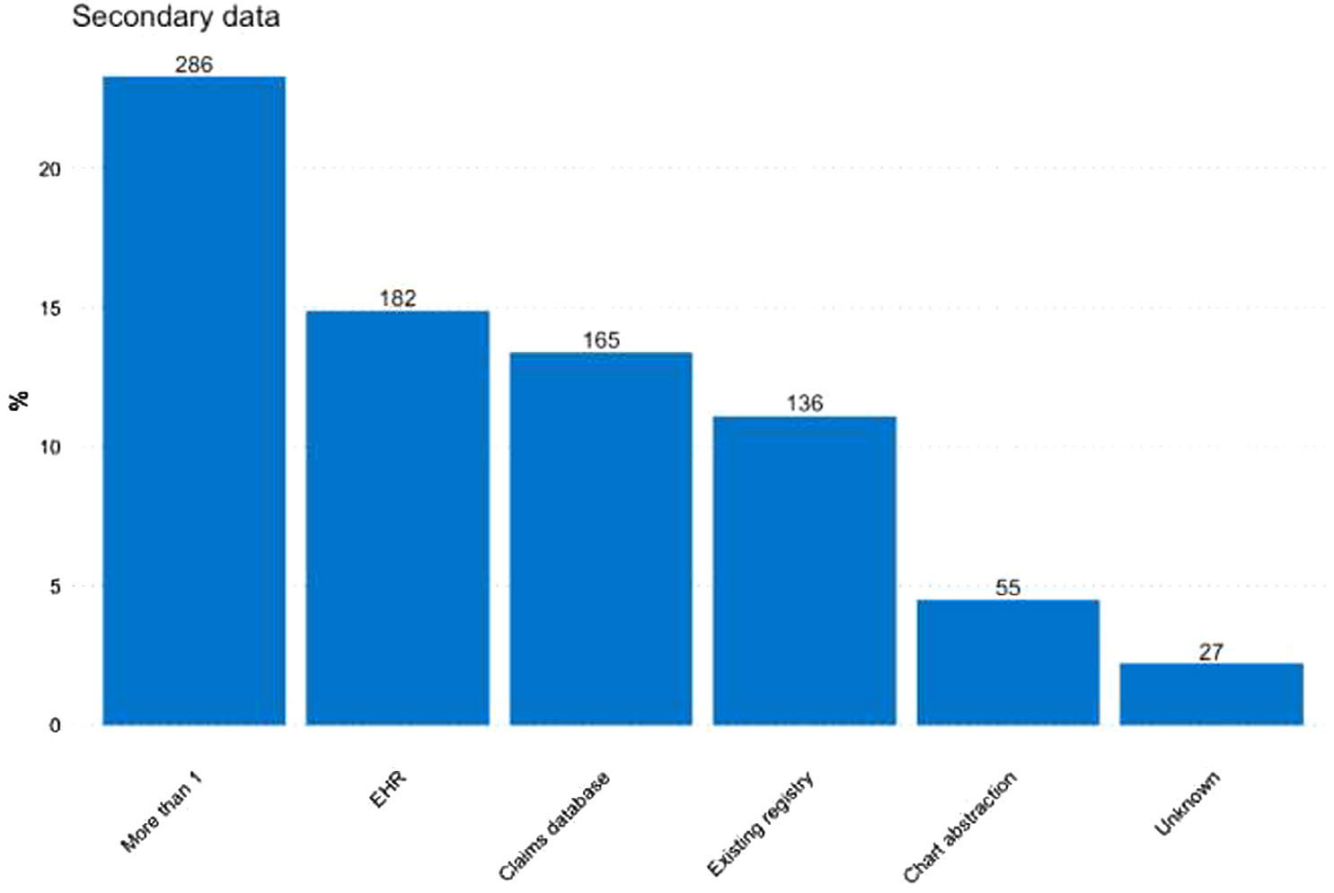
Distribution of different types of secondary data among all observational studies. EHR, electronic health records

**FIGURE 5:**
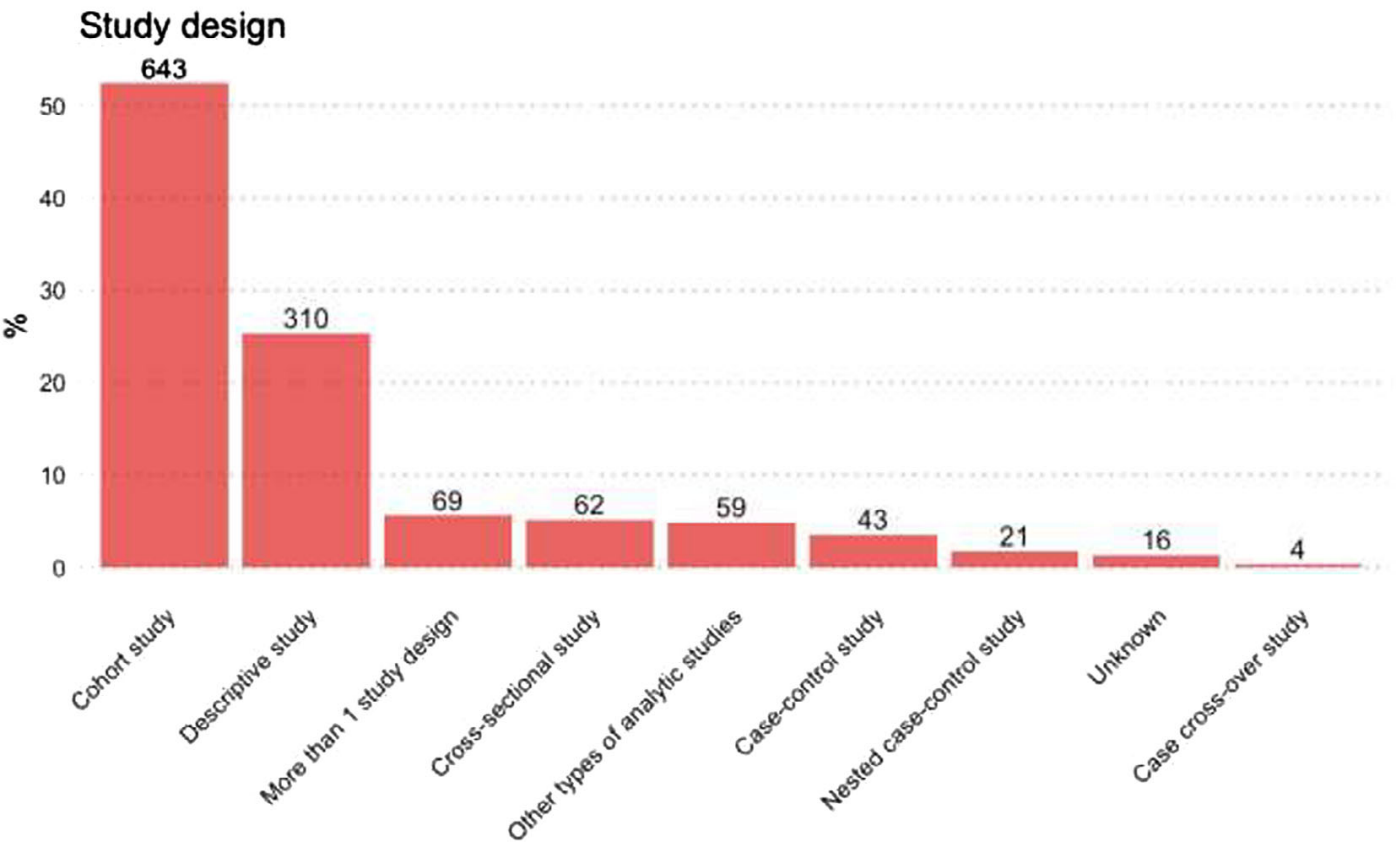
Different types of study designs among observational studies

Concerning the type of secondary data used, among observational studies the use of claims, EHRs or existing registries was similar, at 13.4, 14.9 and 11.1%, respectively (Figure 4). The most commonly used study design among observational studies was the cohort study (52.4%), followed by other types of descriptive studies (23.5%), such as drug utilisation studies, disease epidemiology and pharmacokinetics (Figure 5). Overall, *N* = 319 (26.0%) of observational studies were classified as MDS.

The overall percentage of agreement in data categorisation among collaborating centres that collected the data concerning study variables was highest for study type (93.5%) and lowest for type of secondary data (67.8%; Table 2). The low level of agreement for secondary data is expected to have an impact on the overall level of agreement. These results were largely in line with total kappa coefficients. The values of Cohen's kappa and the centre variations in Cohen's kappa for key, along with their 95% CIs, are shown in Figures S1B1 and S1B2, respectively.

**TABLE 2:** Inter‐rate reliability agreement among collaborating centres in data categorisation

Variables	Categories	Kappa coefficient^a^	Agreement *N* = 214 (%)	Total kappa coefficient^a^	Interpretation of total kappa coefficient
Study type	Clinical trials	0.795	200 (93.5)	0.765	Substantial agreement
	Observational studies	0.758			
	Systematic reviews/meta‐analyses	1.000			
	Questionnaire‐based surveys	0.769			
	Others	0.795			
	Unknown	‐			
Data Collection	Primary	0.717	171 (79.9)	0.652	Substantial agreement
	Secondary	0.666			
	Primary and secondary (mixed)	0.562			
	Unknown	‐			
Drug type	Non‐biologic	0.685	176 (82.2)	0.668	Substantial agreement
	Biologic	0.827			
	Both biologic and non‐biologic	‐			
	None	0.497			
	Unknown	‐			
Use of reference drug for formal comparison	Yes	0.659	171 (79.9)	0.663	Substantial agreement
	No	0.621			
	Unknown	0.127			
Setting	Routine	0.493	193 (90.2)	0.518	Moderate agreement
	Experimental	0.829			
	Unknown	‐			
	Not applicable	0.509			
Secondary data	Chart abstraction	0.481	145 (67.8)	0.501	Moderate agreement
	Claims database	0.131			
	EHR	0.457			
	Existing registry	0.505			
	Not applicable‐not secondary data	0.728			
	More than 1	0.579			
	Unknown	0.314			
Multiple database study	Yes	0.503	176 (77.6)	0.485	Moderate agreement
	No	0.478			
	Unknown	0.274			
Orphan drug	Yes	0.478	179 (83.6)	0.453	Moderate agreement
	No	0.422			
	Unknown	‐			

*Note*: ‐, empty cell in the dataset.

a Kappa result be interpreted as follows: ≤0 no agreement and 0.01–0.20 as none to slight, 0.21–0.40 as fair, 0.41–0.60 as moderate, 0.61–0.80 as substantial, and 0.81–1.00 as almost perfect agreement.^8^

Compared to studies based on the secondary use of data, those based on primary data collection were less likely to have a protocol deposited, to be funded by public entities and to use a reference drug for formal comparison, while they were more likely to be funded by pharmaceutical companies (Table 3). In general, there was no substantial difference in study design between studies based on primary data collection or secondary use of data, although descriptive studies were slightly more common in the former.

**TABLE 3:** Description of the observational studies identified in the EU PAS register. Secondary data was considered the comparator

	Primary and secondary data (mixed) *N* = 147 (%)	Primary data *N* = 299 (%)	Secondary data *N* = 757 (%)	Primary data versus Secondary data OR (95% CI)
Protocol deposited				
Yes	95 (64.6)	146 (48.8)	476 (62.9)	0.5 (0.4–0.7)
No	52 (35.4)	153 (51.2)	281 (37.1)	‐
Source of funding				
Pharmaceutical company	125 (85.0)	272 (91.0)	587 (77.5)	2.9 (1.9–4.4)
National/international drug agency	4 (2.7)	5 (1.7)	43 (5.7)	0.3 (0.1–0.7)
Public entities excluding drug agencies	9 (6.1)	7 (2.3)	48 (6.3)	0.3 (0.1–0.8)
Self‐funded	1 (0.7)	5 (1.7)	17 (2.2)	0.7 (0.2–2.0)
More than one source	8 (5.4)	8 (2.7)	45 (5.9)	0.4 (0.2–0.9)
Unknown	0 (0.0)	2 (0.7)	17 (2.2)	0.2 (0.0–1.2)
RMP status				
Not applicable	59 (40.1)	133 (44.5)	398 (52.6)	0.7 (0.5–0.9)
EU RMP 1	8 (5.4)	24 (8.0)	49 (6.5)	1.2 (0.7–2.1)
EU RMP 2	4 (2.7)	11 (3.7)	15 (2.0)	1.8 (0.8–4.1)
EU RMP 3	52 (35.4)	77 (25.8)	222 (29.3)	0.8 (0.6–1.1)
Non‐EU RMP only	17 (11.6)	42 (14.0)	41 (5.4)	2.8 (1.8–4.4)
Missing—no info at all	7 (4.8)	12 (4.0)	32 (4.2)	0.9 (0.4–1.8)
Study design				
Cohort study	65 (44.2)	156 (52.2)	409 (54.0)	0.9 (0.7–1.2)
Cross‐sectional study	7 (4.8)	20 (6.7)	31 (4.1)	1.6 (0.9–2.9)
Case–control study	7 (4.8)	7 (2.3)	28 (3.7)	0.6 (0.2–1.4)
Case cross‐over study	0 (0.0)	3 (1.0)	1 (0.1)	‐
Nested case–control study	0 (0.0)	1 (0.3)	20 (2.6)	0.1 (0.0–0.9)
Other type of descriptive studies	43 (29.3)	89 (29.8)	172 (22.7)	1.4 (1.0–1.9)
Other types of analytic studies	7 (4.8)	15 (5.0)	37 (4.9)	1.0 (0.5–1.9)
More than 1 study design	15 (10.2)	2 (0.7)	52 (6.9)	0. (0.0–0.3)
Unknown	3 (2.0)	6 (2.0)	7 (0.9)	‐
Use of reference drug for formal comparison				
Yes	32 (21.8)	46 (15.4)	255 (33.7)	0.3 (0.2–0.5)
No	110 (74.8)	242 (80.9)	475 (62.7)	‐
Unknown	5 (3.4)	11 (3.7)	27 (3.6)	‐
Setting				
Routine	140 (95.2)	282 (94.3)	740 (97.8)	0.4 (0.2–0.7)
Experimental	3 (2.0)	7 (2.3)	11 (1.5)	1.6 (0.6–4.2)
Unknown	2 (1.4)	9 (3.0)	1 (0.1)	‐
Not applicable	2 (1.4)	1 (0.3)	5 (0.7)	‐
Scope of the study				
Disease epidemiology	38 (25.9)	40 (13.4)	130 (17.2)	0.7 (0.5–1.1)
Risk assessment	96 (65.3)	178 (59.5)	404 (53.4)	1.3 (0.9–1.6)
Drug utilisation	48 (32.7)	99 (33.1)	287 (37.9)	0.8 (0.6–1.0)
Effectiveness evaluation	54 (36.7)	131 (43.8)	176 (23.2)	2.5 (1.9–3.4)
Other*	34 (23.1)	74 (24.7)	136 (18.0)	1.5 (1.1–2.1)
Population of interest—age				
Children	62 (42.2)	101 (33.8)	272 (35.9)	0.9 (0.7–1.2)
Adults	130 (88.4)	273 (91.3)	679 (89.7)	1.2 (0.7–1.9)
Elderly persons	110 (74.8)	246 (82.3)	634 (83.8)	0.9 (0.6–1.2)
Unknown	‐	‐		‐
Population of interest—special populations				
Immunocompromised	7 (4.8)	27 (9.0)	51 (6.7)	1.3 (0.8–2.2)
Hepatic impairment	11 (7.5)	24 (8.0)	57 (7.5)	1.1 (0.6–1.7)
Renal impairment	8 (5.4)	25 (8.4)	71 (9.4)	0.9 (0.5–1.4)
Pregnant women	21 (14.3)	32 (10.7)	76 (10.0)	1.1 (0.7–1.6)
Breast‐feeding women	0 (0.0)	1 (0.3)	11 (1.5)	0.2 (0.0–1.7)
Other	11 (7.5)	22 (7.4)	39 (5.2)	1.4 (0.8–2.5)
Drug of interest—general				Biologic (vs. all the other categories)
Non‐biologic	76 (51.7)	172 (57.5)	495 (65.4)	1.9 (1.4–2.6)
Biologic	50 (34.0)	94 (31.4)	145 (19.2)	‐
Both biologic and non‐biologic	5 (3.4)	6 (2.0)	14 (1.8)	‐
None	13 (8.8)	19 (6.4)	73 (9.6)	‐
Unknown	3 (2.0)	8 (2.7)	30 (40.0)	‐
Drug of interest—orphan drugs				Orphan drug (vs. all the others)
Yes	21 (14.3)	54 (18.1)	78 (10.3)	1.9 (1.3–2.8)
No	125 (85.0)	233 (77.9)	646 (85.3)	
Unknown	1 (0.7)	12 (4.0)	33 (4.4)	
Publication available				
Yes	51 (34.7)	58 (19.4)	247 (32.6)	0.5 (0.4–0.7)
No	96 (65.3)	241 (80.6)	510 (67.4)	

Abbreviations: 95% CI, 95% confidence interval; PASs, European post‐authorization studies; OR, odds ratio; RMP, risk management plan.

Only a third of all claims‐ and EHR‐based observational studies were requested by a regulator (Table 4). Claims data were more commonly used for risk assessment than EHRs (60.8% vs. 40.7%).

**TABLE 4:** Description of specific types of mixed and secondary data

	Chart abstraction *N* = 63 (%)	Claims database *N* = 171 (%)	EHRs *N* = 189 (%)	Existing registry *N* = 166 (%)	More than one type of data *N* = 290 (%)
	*N* (%)	*N* (%)	*N* (%)	*N* (%)	*N* (%)
Protocol deposited					
Yes	36 (57.1)	84 (49.1)	126 (66.7)	103 (62.0)	209 (72.1)
No	27 (42.9)	87 (50.9)	63 (33.3)	63 (38.0)	81 (27.9)
Requested by a regulator					
Yes	32 (51.1)	55 (32.2)	64 (33.9)	67 (46.4)	150 (51.7)
No	31 (49.2)	115 (67.3)	119 (63.0)	86 (51.8)	133 (45.9)
Unknown	0 (0.0)	1 (0.6)	2 (3.2)	3 (1.8)	7 (2.4)
Source of funding					
Pharmaceutical company	56 (88.9)	134 (78.4)	138 (73.0)	135 (81.3)	218 (75.2)
National/international drug agency	2 (3.2)	10 (5.8)	17 (9.0)	9 (5.4)	12 (4.1)
Public entities excluding drug agencies	4 (6.3)	12 (7.0)	10 (5.3)	5 (3.0)	28 (9.7)
Self‐funded	1 (1.6)	5 (2.9)	2 (1.1)	3 (1.8)	7 (2.4)
More than one source	0 (0.0)	7 (4.1)	15 (7.9)	12 (7.2)	20 (6.9)
Unknown	0 (0.0)	3 (1.8)	7 (3.7)	2 (1.2)	5 (1.7)
RMP status					
Not applicable	28 (44.4)	108 (63.2)	122 (64.6)	73 (44.0)	122 (42.1)
EU RMP 1	9 (14.3)	4 (2.3)	12 (6.3)	10 (6.0)	20 (6.9)
EU RMP 2	1 (1.6)	1 (0.6)	2 (1.1)	9 (5.4)	5 (1.7)
EU RMP 3	18 (28.6)	41 (24.0)	31 (16.4)	49 (29.5)	114 (39.3)
Non‐EU RMP only	3 (4.8)	14 (8.2)	13 (6.9)	14 (8.4)	15 (5.2)
Missing—no info at all	4 (6.3)	3 (1.8)	9 (4.8)	11 (6.6)	14 (4.8)
Data model					
Local data extraction and analysis, common protocol	‐	‐	‐	‐	‐
Local data extraction and central analysis on patient‐level raw data	‐	‐	‐	‐	‐
Study‐specific local data extraction in a common data model and central analysis	‐	‐	‐	‐	‐
General local data extraction in a common data model and central analysis	‐	‐	‐	‐	‐
Not applicable	36 (57.1)	109 (63.7)	122 (64.6)	93 (56.0)	54 (18.6)
Unknown	10 (15.9)	21 (12.3)	15 (7.9)	17 (10.2)	77 (26.6)
Missing	11 (17.5)	29 (17.0)	16 (8.5)	41 (24.7)	57 (19.7)
Study design					
Cohort study	22 (34.9)	109 (63.7)	90 (47.6)	83 (50.0)	154 (53.1)
Cross‐sectional study	5 (7.9)	1 (0.6)	12 (6.3)	8 (4.8)	10 (3.4)
Case–control study	1 (1.6)	6 (3.5)	8 (4.2)	10 (6.0)	10 (3.4)
Case cross‐over study	0 (0.0)	0 (0.0)	0 (0.0)	1 (0.6)	0 (0.0)
Nested case–control study	0 (0.0)	4 (2.3)	8 (4.2)	1 (0.6)	8 (2.8)
Other types of descriptive studies	23 (36.5)	29 (17.0)	50 (27.5)	39 (23.5)	63 (21.7)
Other types of analytic studies	7 (11.1)	3 (1.8)	11 (5.8)	15 (9.0)	9 (3.1)
More than 1 study design	3 (4.8)	16 (9.4)	8 (4.2)	8 (4.8)	31 (10.7)
Unknown	2 (3.2)	3 (1.8)	0 (0.0)	1 (0.6)	5 (1.7)
Use of reference drug for formal comparison					
Yes	7 (11.1)	86 (50.3)	47 (24.9)	45 (27.1)	97 (33.4)
No	52 (82.5)	73 (42.7)	138 (73.0)	114 (68.7)	187 (64.5)
Unknown	4 (6.3)	12 (7.0)	4 (2.1)	7 (4.2)	6 (2.1)
Scope of the study					
Disease epidemiology	6 (9.5)	26 (15.2)	36 (19.0)	44 (26.5)	49 (16.9)
Risk assessment	28 (44.4)	104 (60.8)	77 (40.7)	95 (57.2)	175 (60.3)
Drug utilisation	31 (49.2)	58 (33.9)	73 (38.6)	50 (30.1)	117 (40.3)
Effectiveness evaluation	25 (39.7)	37 (21.6)	54 (28.6)	43 (25.9)	54 (18.6)
Other*	17 (27.0)	31 (18.1)	31 (16.4)	33 (19.9)	53 (18.3)
Population of interest—age					
Children	15 (23.8)	42 (24.6)	60 (31.7)	67 (40.4)	133 (45.9)
Adults	58 (92.1)	148 (86.5)	176 (93.1)	147 (88.26	256 (88.3)
Elderly persons	54 (85.7)	144 (84.2)	160 (84.7)	127 (76.5)	240 (82.8)
Population of interest—special populations					
Immunocompromised	2 (3.2)	8 (4.7)	11 (5.8)	21 (12.7)	16 (5.5)
Hepatic impairment	3 (4.8)	8 (4.7)	14 (7.4)	21 (12.7)	23 (7.9)
Renal impairment	4 (6.3)	13 (7.6)	13 (6.9)	22 (13.3)	26 (9.0)
Pregnant women	2 (3.2)	18 (10.5)	14 (7.4)	29 (17.5)	33 (11.4)
Breast‐feeding women	0 (0.0)	1 (0.6)	0 (0.0)	4 (2.4)	6 (2.1)
Other	5 (7.9)	12 (7.0)	13 (6.9)	5 (3.0)	11 (3.8)
Drug of interest—general					
Non‐biologic	30 (47.6)	116 (67.8)	130 (68.8)	91 (54.8)	189 (65.2)
Biologic	25 (39.7)	33 (19.3)	35 (18.5)	39 (23.5)	57 (19.7)
Both biologic and non‐biologic	2 (3.2)	3 (1.8)	1 (0.5)	7 (4.2)	5 (1.7)
None	3 (4.8)	12 (7.0)	18 (9.5)	22 (13.3)	29 (10.0)
Unknown	3 (4.8)	7 (4.1)	5 (2.6)	7 (4.2)	10 (3.4)
Drug of interest—orphan drugs					
Yes	8 (12.7)	14 (8.2)	16 (8.5)	31 (18.7))	20 (6.9)
No	53 (84.1)	147 (86.0)	168 (88.9)	129 (77.7)	258 (89.0)
Unknown	2 (3.2)	10 (5.8)	5 (2.6)	6 (3.6)	12 (4.1)
Publication available					
Yes	19 (30.2)	63 (36.8)	73 (38.6)	45 (27.1)	94 (32.4)
No	44 (69.8)	108 (63.2)	116 (61.4)	121 (72.9)	196 (67.6)

Abbreviations: EHR, electronic healthcare record; RMP, risk management plan.

Among observational studies, as compared to non‐MDS, MDS were more likely to have a protocol deposited in the EU PAS register (OR: 1.9; 95% CI: 1.4–2.5), to have an ENCePP Seal (OR: 3.0; 1.9–4.7), to be funded by national/international drug agencies (OR: 1.9; 95% CI: 1.1–3.4) and to include children as study population (OR: 2.1; 95% CI: 1.5–2.6). Moreover, almost two‐thirds of MDS were requested by a regulator. Interestingly, drug utilisation was significantly more common as a study scope in MDS compared to non‐MDS studies (OR: 1.3; 95% CI: 1.1–1.7). However, MDS were less likely to consider orphan drugs as the main exposure (OR: 0.5; 95% CI: 0.3–0.8) (Table 5).

**TABLE 5:** Multiple database studies versus non‐multiple database studies. MSD was considered the comparator

	MDS *N* = 319 (%)	Non‐MDS *N* = 864 (%)	OR (95% CI)
Protocol deposited			
	222 (69.6)	492 (56.9)	1.9 (1.4–2.5)
EnCePP seal			
	42 (13.4)	43 (4.9)	3.0 (1.9–4.7)
Requested by a regulator			
	184 (57.7)	372 (43.0)	1.6 (1.2–1.9)
Status			
Planned	48 (15.3)	115 (13.4)	1.2 (0.8–1.7)
Ongoing	114 (36.5)	382 (44.2)	0.7 (0.5–0.8)
Finalised	157 (50.3)	367 (42.4)	1.3 (1.1–1.7)
Source of funding			
Pharmaceutical company	250 (78.4)	715 (82.7)	0.7 (0.5–1.1)
National/international drug agency	21 (6.6)	31 (3.6)	1.9 (1.1–3.4)
Public entities excluding drug agencies	20 (6.3)	44 (5.1)	1.3 (0.7–2.2)
More than one source	5 (1.6)	42 (4.8)	1.3 (0.7–2.2)
Self‐funded	19 (6.0)	17 (1.9)	0.8 (0.3–2.1)
Unknown	4 (1.3)	15 (1.7)	0.7 (0.2–2.3)
PI employed by study funder			
	170 (53.3)	560 (64.8)	0.6 (0.5–0.8)
RMP status			
EU RMP 1	134 (42.0)	50 (5.8)	1.7 (1.1–2.7)
EU RMP 2	30 (9.4)	24 (2.7)	0.7 (0.3–1.7)
EU RMP 3	6 (1.9)	225 (26.0)	1.8 (1.4–2.3)
Non‐EU RMP only	123 (38.6)	82 (9.5)	0.5 (0.3–0.8)
Not applicable	15 (4.7)	442 (51.1)	0.7 (0.5–0.9)
Missing—no info at all	11 (3.4)	41 (4.7)	0.7 (0.4–1.4)
Data collection			
Primary	‐	298 (34.5)	‐
Secondary	282 (90.3)	452 (52.3)	6.9 (4.8–10.1)
Mixed	35 (11.2)	108 (12.5)	0.9 (0.6–1.3)
Unknown	2 (0.6)	6 (0.7)	‐
Secondary data			
Claims database	26 (8.3)	132 (15.2)	0.5 (0.3–0.7)
Product lifecycle			
Post‐marketing	2 (0.6)	795 (92.0)	0.9 (0.6–1.4)
Not applicable	291 (91.2)	60 (7.0)	0.8 (0.4–1.3)
Pre‐marketing	18 (5.6)	2 (0.2)	‐
Unknown	8 (2.5)	8 (0.9)	‐
Study design			
Cohort study	76 (23.8)	456 (52.8)	1.1 (0.8–1.3)
Case–control study	168 (52.7)	33 (3.8)	0.7 (0.3–1.6)
Case cross‐over study	13 (4.1)	4 (0.5)	‐
Cross‐sectional study	9 (2.8)	45 (5.2)	0.7 (0.4–1.4)
Nested case–control study	0 (0.0)	15 (1.7)	1.1 (0.4–2.9)
Other types of analytic studies	6 (1.9)	44 (5.1)	0.8 (0.4–1.5)
Other types of descriptive studies	13 (4.1)	222 (25.7)	0.9 (0.7–1.2)
More than 1 study design	32 (10.0)	34 (3.9)	2.6 (1.6–4.3)
Unknown	2 (0.6)	11 (1.3)	‐
Use of reference drug for formal comparison			
	102 (32.7)	224 (25.9)	1.3 (0.9–1.7)
Setting			
Routine	308 (96.6)	835 (86.9)	1.1 (0.6–2.3)
Experimental	7 (2.2)	13 (1.5)	1.4 (0.6–3.6)
Not applicable	2 (0.6)	6 (0.7)	0.9 (0.2–4.7)
Unknown	2 (0.6)	10 (1.2)	‐
Scope of the study			
Disease epidemiology	58 (18.2)	145 (16.8)	1.1 (0.8–1.5)
Risk assessment	187 (58.6)	488 (56.5)	1.1 (0.8–1.4)
Drug utilisation	132 (41.4)	295 (34.1)	1.3 (1.1–1.7)
Effectiveness evaluation	55 (17.2)	297 (34.4)	0.4 (0.3–0.5)
Other	52 (16.3)	188 (21.8)	0.7 (0.5–1.1)
Population of interest—age			
Children	154 (48.3)	284 (32.9)	2.1 (1.5–2.6)
Adults	286 (89.7)	776 (89.8)	0.9 (0.6–1.4)
Elderly persons	266 (83.4)	703 (81.4)	1.1 (0.8–1.6)
Unknown	3 (0.9)	8 (0.9)	1.1 (0.3–4.1)
Population of interest—special populations			
Immunocompromised	22 (6.9)	64 (7.4)	0.9 (0.6–1.6)
Hepatic impairment	26 (8.2)	67 (7.8)	1.1 (0.7–1.7)
Renal impairment	28 (8.8)	76 (8.8)	0.9 (0.6–1.6)
Pregnant women	37 (11.6)	93 (10.8)	1.1 (0.7–1.7)
Breast‐feeding women	6 (1.9)	7 (0.8)	2.5 (0.8–7.3)
Other	14 (4.4)	57 (6.6)	0.6 (0.4–1.2)
Drug of interest (general)			
Biologic (vs. all the others)	66	270	0.7 (0.6–1.1)
Orphan drug (vs. all the others)	26	149	0.5 (0.3–0.8)
Publication available			
	101 (31.7)	250 (28.9)	1.2 (0.9–1.5)

Abbreviations: 95% CI, 95% confidence intervals; MDS, multiple database study; OR, odds ratio.

## 4 DISCUSSION

The present study provides a detailed description of all studies registered in the EU PAS register from its inception until the end of 2018, focusing and providing detail on various aspects of study design and multiple database studies specifically. As expected, as compared to the most recent review of EU PAS register available,^4^ our results showed a steady increase in the number of observational studies registered. The present study adds to the available literature by providing the most recent updated review of the EU PAS register, including all PAS rather than just PASS. To do this, we leveraging a network of pharmacoepidemiologists from various centres belonging to ENCePP to collect additional and detailed information on methodological aspects of observational studies. Furthermore, to our knowledge, the present study is the only one that validates the inter‐rater reliability of different collaborating centres during the data collection process, thus providing indirect measure of the completeness and accuracy of the data collected in the EU PAS register.

From our study, it appears that only 61.7% of observational studies were conducted through the secondary use of existing healthcare data. This finding indicates that the remaining one third of observational studies were conducted using the much more resource‐intensive method of collecting data ad hoc, that is, primary data collection. It is not known whether such studies could have been easily conducted using routinely collected healthcare data, which provide detailed information on population characteristics, exposure and outcome. In general, we expected fewer studies to have been conducted using primary data, as large use of primary data source can reflect the need to improve the access to data for research purpose. In line with previous studies, primary data were more commonly used for effectiveness and risk assessment studies,^3^, ^4^ while secondary data was more likely to be used for drug utilisation studies and risk assessment studies. The increase in use secondary data source was sharpest in the last 2 years, while until 2016 the use of primary data source was in in 56% of the 316 studies identified in the EU PAS register.^4^

Of note, some markers of high ethical standards and transparency such as protocol deposition are often missing. This result has to be balanced with the fact that registration is legally binding and subject to financial penalties only for imposed PASS at the time of final study report. Finalised studies that are RMP 1 and 2 with protocol available were 68.5%. It was found that 37% of clinical trials did not have a deposited protocol while 58% of observational trials did. Of 1204 observational studies, 15 (1.2%) had no information whatsoever on study design, highlighting the importance of transparency. Transparency in research along with methodological rigour are essential to increase the standards of pharmacoepidemiological research in Europe.^9^ Competent authorities should keep emphasising the importance of study registration in order to increase the level of transparency in the research landscape, particularly for publically funded research. Another measure of high ethical standards and transparency, the ENCePP Seal, was only available in 7% of all observation studies. The ENCePP Seal identifies studies in the register that adhere to the entirety of the Code's provisions, including timelines for the submission of documents to the ENCePP Secretariat. However, obtaining this Seal is associated with an added bureaucratic burden that researchers may not be willing to undertake. The usefulness of the ENCePP Seal on a broader scale may be somewhat limited if so few studies undertake the procedures to obtain it. Other initiatives such as the revision of the ENCePP Code of Conduct have recently been undertaken but it is not known to what extent this is being adopted.^10^

Another interesting finding of this study with important implications concerns the level of agreement seen between pharmacoepidemiologists in the process of data collection from the EU PAS register. While there was substantial agreement for study type, data collection, drug type and use of reference drug for formal comparison, there was only moderate agreement for the rest of the fields, including study setting, classification of secondary data, whether the study was an MDS and whether the study drug was an orphan drug. High agreement was not reached for any of the fields, due to ambiguous, missing or conflicting reporting. These findings are thought provoking and suggest that data in the EU PAS register was not always clear and complete. Indeed, the quality of data entry in the EU PAS register is not monitored at all as with other similar platforms such as clinicaltrials.gov. In the latter, National Library of Medicine (NLM) staff conducts a review of registered study records for obvious errors or inconsistencies, with important issues being communicated directly to the investigators.^11^ However, this does not guarantee complete and accurate records for all studies.^12^ In the EU PAS register, it is completely up to the investigator's discretion to conduct data entry correctly and accurately as no quality control is conducted. The very small number of duplicate studies identified, two, is potentially an indicator that in this sense, the register's data is of satisfactory quality. In addition, there are several open text fields, missing categories in the different attributes and certain categories used in more than one attribute which makes it challenging to identify and summarise study types in the EU PAS register information. It should be noted that there is currently no glossary in the EU PAS register to promote harmonisation of terms used. We argue that the terminology to be used should be based on a single accepted definition that is presented in a glossary. The reason is that the classification of studies and other relevant aspects (e.g. data sources, methods etc.) was often found to be conducted using ambiguous or even incorrect terms. For the EU PAS register to hold promise of a greater transparency and accuracy, by serving as a common repository to share pharmacoepidemiological research, it would be key to further standardise its data elements, and create completion guidelines to offer an unequivocal interpretation of the fields by study registrants.

With our working definition of MDS as studies which use more than one secondary data source, we found more than 20% of observational studies classified as MDS. These studies were more likely to have the ENCePP Seal and to have protocol deposited, all markers of high transparency. Orphan drugs were not commonly investigated using MDS, although such studies may have a lot of potential in the rare disease field given the small population sizes expected and the increasing need of merging data from different sources to speed up the development of these medicines.

Our analysis has several strengths compared with previous studies as it provides a detailed overview of the studies registered on EU PAS register, with systemic data collection being conducted by a network of established centres of excellence in pharmacoepidemiology. It is very important to underline that data collection was carried out by researchers independently based on common and very detailed instructions. These instructions were shared, agreed and made accessible and available among all reviewers, to avoid potential errors in the classification method. Again, to further check, blind quality control of data entry was conducted for some fields, to obtain an overall agreement. In addition, this is the first time that an evaluation of the MDS landscape has been attempted.

However, this study also has some limitations. The most important limitation concerns the accuracy of the data collection, which was sometimes limited by the lack of information recorded in the EU PAS register or lack of clarity of such information. The lack of data and ambiguities in classification of some studies may limit the robustness of our analyses. In these instances, the judgement on categorising characteristics of a study was somewhat subjective. This is a limitation inherent to the way that data is collected in the EU PAS register and to the lack of quality control of such data. Furthermore, since the registration of a study is voluntary, the EU PAS register is not representative of the whole landscape of studies in Europe and some studies may have not been captured. Another limitation is that the data was collected only until 2018. However, we hold that the present study still has the added value of providing an overview of the EU PAS register compared to the most recently published reviews, which described studies until 2016.^4^

## 5 CONCLUSION

Observational studies were the most common type of studies in the EU PAS register, but almost one third of observational studies used primary data, which is more resource‐intensive. One quarter of observational studies were MDS. MDS hold untapped potential to investigate special populations, such as patients with rare diseases and paediatric patients. A detailed analysis of IRR during data collection suggested that information recorded in the EU PAS register is often not clear or complete. Increasing the quality of study‐related information would considerably increase the transparency of research.

## CONFLICT OF INTEREST

The authors declare no conflicts of interest.

## ETHICS STATEMENT

For this study, there was no need to set up or directly manage databases in which there might have been identifiable or identified patient data; therefore, according to local regulations, obtaining an ethics committee approval to carry out this study was not necessary.

## Supporting information


**Appendix A**: Study protocol

**Appendix Figure B1**: Cohen's Kappa for key variables with 95% confidence intervals.

**Appendix Figure B2**: Centre variations in Cohen's Kappa for key variables with 95% confidence intervals.

## References

[1] European Network of Centres for Pharmacoepidemiology and Pharmacovigilance . The European Union electronic Register of Post‐Authorisation Studies (EU‐PAS Register). Accessed August 31, 2021. http://www.encepp.eu/encepp_studies/indexRegister.shtml

[2] European Medicines Agency . GVP Module VIII; 2017. Accessed August 31, 2021. https://www.ema.europa.eu/en/documents/scientific-guideline/guideline-good-pharmacovigilance-practices-gvp-module-viii-post-authorisation-safety-studies-rev-3_en.pdf

[3] Engel P , Almas MF , De Bruin ML , Starzyk K , Blackburn S , Dreyer NA . Lessons learned on the design and the conduct of post‐authorization safety studies: review of 3 years of PRAC oversight. Br J Clin Pharmacol. 2017;83(4):884‐893. 27780289 10.1111/bcp.13165 PMC5346857

[4] Carroll R , Ramagopalan SV , Cid‐Ruzafa J , Lambrelli D , McDonald L . An analysis of characteristics of post‐authorisation studies registered on the ENCePP EU‐PAS register. F1000Res. 2017;6:1447. doi:10.12688/f1000research.12198.2 29188016 PMC5698914

[5] Vora P , Artime E , Soriano Gabarró M , Qizilbash N , Singh V , Asiimwe A . A review of studies evaluating the effectiveness of risk minimisation measures in Europe using the European Union electronic register of post‐authorization studies. Pharmacoepidemiol Drug Saf. 2018;27:695‐706. doi:10.1002/pds.443 29663572 PMC6055865

[6] Farcas A , Huruba M , Mogosan C . Study design process and outcome indicators of post‐authorization studies aimed at evaluating the effectiveness of risk minimization measures in the EU‐PAS register. Br J Clin Pharmacol. 2019;85(3):476‐491. doi:10.1111/bcp.13824 30497102 PMC6379233

[7] Gini R , Sturkenboom MCJ , Sultana J , et al. Different strategies to execute multi‐database studies for medicines surveillance in real‐world setting: a reflection on the European model. Clin Pharmacol Ther. 2020;108(2):228‐235. 32243569 10.1002/cpt.1833 PMC7484985

[8] McHugh ML . Inter‐rater reliability: the kappa statistic. Biochem Med (Zagreb). 2012;22(3):276‐2829. 23092060 PMC3900052

[9] Blake KV , Prilla S , Accadebled S , et al. European medicines agency review of post‐authorisation studies with implications for the European network of Centres for Pharmacoepidemiology and Pharmacovigilance. Pharmacoepidemiol Drug Saf. 2011;20(10):1021‐1029. doi:10.1002/pds.2209 22039593

[10] Gini R , Fournie X , Dolk H , et al. The ENCePP code of conduct: a best practise for scientific independence and transparency in noninterventional postauthorisation studies. Pharmacoepidemiol Drug Saf. 2019;28(4):422‐433. 30838708 10.1002/pds.4763 PMC6594014

[11] Clinicaltrials.gov . Glossary of Common Site Terms; 2021. Accessed August 31, 2021. https://clinicaltrials.gov/ct2/about-studies/glossary

[12] Miron L , Gonçalves RS , Musen MA . Obstacles to the reuse of study metadata in ClinicalTrials.gov. Sci Data. 2020;7:443. 33339830 10.1038/s41597-020-00780-z PMC7749162

