# Supplementary material for: Overview of the European post‐authorisation study register post‐authorization studies performed in Europe from September 2010 to December 2018
Source: Pharmacoepidemiol Drug Saf. 2022 Feb 11;31(6):689–705. doi: 10.1002/pds.5413 (PMC9303697; doi:10.1002/pds.5413)
Supplement: Supplementary file 1 — Appendix A: Study protocol [file PDS-31-689-s002.docx]

**Appendix A : Study protocol**

**EU-PAS Register studies classification instructions**

**Assigned Studies**

Please find your centre’s name in the data collection file, from the filter **Assigned Centre**, select your centre. For all centres which have specified which reviewers will be participating, a specific reviewer is assigned in the column **Assigned Person**. For those centres who did not specify all the participants, the Assigned person reads “**To be assigned internally**”.

**Introduction**

The contents of data collection form are currently broadly divided into:

1. Automatically extracted data: **this may need to be updated, as data is correct at January 2019 and may have changed since then; automatically extracted data should also be checked for correctness and if needed, modified.**

2. Data requiring manual extraction

**Study selection**

The studies were included based on their “creation date”, which should be at most 31^st^ December 2018. The “last updated” field was not used, as this would lead to an exclusion of studies which were registered several years ago but were updated in January 2019 onwards.

**Data collection**

**Priority should be given to information in the English version of the study protocol uploaded on the EU-PAS register study page,** with as much information as possible being derived from it. In case of conflicting information in the protocol and in the EU-PAS register website, **priority should be given to the protocol**.

In the instructions below, the fields requiring manual data collection are accompanied by specific suggestions of where to find the information directly or to deduce it using the EU-PAS Register form. However, all reviewers are **strongly** encouraged to read all the EU-PAS registry form in addition to the protocol for each study very carefully, as information is not always recorded in a systematic and clear way.

Any difficulties in data collection that are encountered should be noted at the end of the data collection form.

**1. Automatically extracted data**

The following fields are **automatically inserted from a dataset sent by EMA but require checking as they may be updated since January 2019 (last data collected)**:

- **EU_PAS_Register_number**: the unique identification number for each study found in **Section 1** of the EU-PAS register form.
- **Title**: Contains the study title.
- **Last_Updated**: This contains the date when the information on a specific study was last updated. All data are correct at 31^st^ January 2019.
- **ENCePP_Seal**: In response to the question “Have studies been granted an ENCePP seal”
  - **Yes;**
  - **No.**
- **Original_study_type**: The original study classification as found in **Section 1** of the EU-PAS register form
  - **Active surveillance;**
  - **Observational study;**
  - **Clinical trial;**
  - **Other.**
- **Study_requested_by_a_regulator**: In response to the question “Was this study requested by a regulator?” in **Section 1** of the EU-PAS Register form
  - **Yes;**
  - **No;**
  - **Don't know.**
- **Status_of_Study**: Available on every tab of the EU-PAS Register online form
  - **Planned;**
  - **Ongoing;**
  - **Finalised.**
- **Funding_manual_check:** A manual check carried out by UniMe to ascertain correctness of automatically extracted data. Based on **Section 4** of the EU-PAS Register form:
  - **Pharmaceutical companies;**
  - **Charities;**
  - **Government body;**
  - **Research councils;**
  - **EU funding scheme;**
  - **More than 1.**

**2. Data requiring manual extraction**

- **Protocol_in_English_available_on_EU_PAS_site:** Based on the availability of a study protocol in English in section 17 of the EU-PAS register; if a protocol is available in a language other than English, please make a note of this at the end of a document as we may decide at a later date to ask a native speaker to revise the protocol in their language. If there are two protocols, please consider the most recent one:
  - **Yes;**
  - **No.**
- **Funding_source_recateg:** An alternative proposal to the automatically extracted information, using information found in the EU-PAS Register, **Section 4:**
  - **Funded by pharmaceutical company**, if based on section 4 the field “pharmaceutical company” is compiled;
  - **Funded by national/international drug agency**, if based on section 4, one the fields “Government body” specifically mentions EMA (Europe) or one of the national drug agencies of the EU (available from this link: <https://www.ema.europa.eu/en/partners-networks/eu-partners/eu-member-states/national-competent-authorities-human>), the FDA or Health Canada. In case of doubt, please flag your difficulty at the end of the Excel file;

- - **Funded by public entities, excluding drug agencies**, if based on section 4;
  - **Self-funded**, if based on section 4 all the available fields for funding information are empty;
  - **More than one**, if based on section 4 there is more than one funding field compiled;
  - **Unknown**, if based on section 4 it is not clear under which of the above categories the study would fall.
- **PI_employed_by_study_funder**: using information in **Section 4** (sources of funding) and **5** (contact details for enquiries):
  - **Yes**, if the principal investigator (PI), i.e. the main contact as per section 5, has the same affiliation as the funder; this can also be identified from the email address that the PI reports;
  - **No**, i.e. the main contact as per section 5, does not have the same affiliation as the funder;
  - **Unknown**, if it is not clear whether the PI is employed by the funder.
- **Risk_Management_Plan:** Based on **section 1** of the online form, in response to the question “Is the study required by a Risk Management Plan (RMP)?” the categories available are:
  - **EU RMP status not applicable - EU risk management plan status not applicable;**
  - **EU RMP 1: EU risk management plan 1 (imposed as condition of marketing authorisation);**
  - **EU RMP 2: EU risk management plan 2 (specific obligation of marketing authorisation);**
  - **EU RMP 3: EU risk management plan 3 (required);**
  - **Non-EU RMP only – Non-EU risk management plan;**
  - **Unknown**, where this field is empty or otherwise unclear;
  - **Not applicable.**
- **EPAR_link_for_RMP_1_and_2**: For studies classified as having RMP 1 and 2, please find the first European public assessment reports (EPAR) link for a specific medicinal product from the following EMA link: <https://www.ema.europa.eu/en/medicines/field_ema_web_categories%253Aname_field/Human/ema_group_types/ema_medicine>. The website should be searched for the drug trade name as reported in the EU-PAS register.
  - **[Insert free-text link]**
- **Data_collection:** Data collection should be classified, using the **protocol** and/or **section 1 (“Brief description of the study”)** and/or **10** of the EU-PAS Register form as
  - **Primary data,** collection if the data analysed are collected *ex novo* by the researcher for a specific research or project purpose. Surveys will be considered as primary data collection;
  - **Secondary data,** collection if the data analysed are not generated *ex novo* for a specific research purpose but collected for administrative reasons or in the management of own clinical practice;
  - **Unknown,** if the type of data used is not clear;
  - **Mixed,** data collection if the data analysed are both primary and secondary.
- **Secondary_data:** The secondary data type should be classified as follows using **the protocol** and/or **section 1 (“Brief description of the study”) and 10** of the EU-PAS Register form**:**
  - **Chart abstraction**, if the study extracts or collects data contained in patient records, e.g. studies that evaluate the occurrence of adverse events in the hospital;
  - **Claims database**, where "claims" means claims/healthcare reimbursement data or other administrative data;
  - **EHR,** where this refers to the systematic collection of electronically stored health information on patients and populations in digital format performed by a health worker during his clinical practice;
  - **Existing registry**, where "registry" means data *already collected* on a population defined by the use of a specific drug and / or by the presence of a specific pathology;
  - **More than 1**, this concerns studies which use more than one type of secondary data source (e.g. EHR and claims together, or EHR and surveys);
  - **Unknown**, if is not possible to classify the “secondary data” used;
  - **Not applicable-not secondary data**, if data collection is primary.
- **Multiple_database_study:** A study which uses more than one *secondary* data source, based on the **protocol** and/or **section 10** of the EU-PAS Register
  - **Yes;**
  - **No;**
  - **Unknown.**
- **Data_models:** The studies will be classified as using one of the following data models
  - **Local data extraction and analysis, common protocol;**
  - **Local data extraction and central analysis on patient-level raw data;**
  - **Study-specific local data extraction in a common data model and central analysis;**
  - **General local data extraction in a common data model and central analysis;**
  - **Not applicable**, if the study in question is not a multiple database;
  - **Unknown**, if it is not clear.

**Study_type_new_classification**, i.e. of the following options using information from the **protocol** and/or **section 10 and 13** of the EU-PAS Register:

- - **Observational study**, defined by ENCePP in 2011 using a different term, “non-interventional study” as “*a study where the medicinal product(s) is (are) prescribed independent to inclusion of the patient in the study and as part of a therapeutic strategy, including diagnostic and monitoring procedures, which is not decided in advance by a study protocol but is applied according to the current clinical practice*”. Source: <http://www.encepp.eu/publications/documents/ENCePPinterpretationofnoninterventionalstudies.pdf>;
  - **Review or meta-analysis** if they concern the collection / processing of studies results already existing in the literature;
  - **Survey**, defined by ENCePP as “*a data collection tool used to gather information about individuals. Surveys are commonly used to collect self-reported data, either on factual information about individuals, or their opinions. They generally have a cross-sectional design and represent a form primary data collection conducted through questionnaires administered by web, phone or paper*”. (Source: <http://www.encepp.eu/standards_and_guidances/methodologicalGuide4_1_1.shtml>) Surveys include surveys addressed to patients, care-givers, clinicians or other persons;
  - **Clinical trial,** defined by the US National Institute of Health as a study “*in which one or more human subjects are prospectively assigned to one or more interventions (which may include placebo or other control) to evaluate the effects of those interventions on health-related biomedical or behavioural outcomes*.” (Source: <https://grants.nih.gov/policy/clinical-trials/definition.htmv>);
  - **Other** if the study type does not fit into any of the above categories, including studies which are not described clearly and use more than 1 study design. This includes studies which are pharmacovigilance studies based on spontaneous safety, post-hoc analysis of clinical trial data etc.;
  - **Unknown**, if it is not clear what study type was used.
- **Product_lifecycle,** i.e. one of the following options using information from the **protocol** and/or **section 1, 6, 10, 13** of the EU-PAS Register:
  - **Pre-marketing (for ANY indication),** if the study is clearly a clinical trial in an experimental setting;
  - **Post-marketing**, if the study is clearly not a clinical trial in an experimental setting; a study evaluating drug use, safety or efficacy conducted using observational data, such as EHRs or claims data, can be assumed to be post-marketing, as such data sources necessarily only capture drugs post-marketing;
  - **Not applicable**, for example if the study is not focused on a drug;
  - **Unknown**, if it is not clear what the stage of the product lifecycle was.

- **Study_design**, using **the protocol** and/or **section 13** of the EU-PAS register form:
  - **Descriptive study**, i.e. studies which do not evaluate an outcome;
  - **Analytic studies-cohort studies,** i.e. as a general rule if the study fits the definition in Begaud’s Dictionary of Pharmacoepidemiology, 3^rd^ Edition: “Epidemiological design in which the population of subjects having presented an event during the follow-up of a cohort is compared, with respect to an exposure at baseline, with a control population chosen at random from the whole population of subjects present at the beginning of this period”; however, studies which consist of a cohort but with no formal comparison, for example, only measuring the incidence of a specific outcome, should also be classified as cohort studies but flagging the column “Use_of_reference_drug_for_formal_comparison” as “No”; studies which contain more than one cohort should be classified as cohort studies;
  - **Analytic studies-cross sectional studies,** i.e. if the study fits the definition in Begaud’s Dictionary of Pharmacoepidemiology, 3^rd^ Edition: “Study in which the prevalence of a variable (e.g., exposure, an event, a disease) is measured in a population at a given moment; this can also be termed prevalence study; more specifically, an analytic cross-sectional study will be defined as one where patient-level data concerning both exposure and outcome is measure cross-sectionally, at a single point in time. Cross-sectional studies which evaluate only exposure OR outcome, but not both, will be considered descriptive studies;
  - **Analytic studies-case control studies,** if the study fits the definition in Begaud’s Dictionary of Pharmacoepidemiology, 3^rd^ Edition: “Epidemiological design comparing previous exposure to a risk factor of interest (e.g., use of a drug) or the presence of a characteristic in a group of subjects presenting a given event (the cases), to that in a group not presenting this event (the controls)”;
  - **Analytic studies-case cross-over studies,** if the study fits the definition in Begaud’s Dictionary of Pharmacoepidemiology, 3^rd^ Edition: “Epidemiological design to evaluate a possible association between an exposure and the occurrence of an event by comparing the number of cases arising within and outside a previously defined window of exposure, in a population whose exposure status changes over time”;
  - **Analytic studies-nested case-control**, if the study fits the definition in Begaud’s Dictionary of Pharmacoepidemiology, 3^rd^ Edition: “Case-control study carried out within the population of a cohort”;
  - **Analytic studies-other**, if the analytic study design does not fit into the above categories;
  - **More than 1**, i.e. more than one study design**;**
  - **Unknown**, if it is not apparent or clear which study design is being implemented, including studies with conflicting information.
- **Use_of_reference_drug_for_formal_comparison**, using information from the **protocol** and/or **section 12, and 15** of the EU-PAS Register and the protocol if present:
  - **Yes**, if the study information indicates that a study drug is compared to an active or inactive comparator to provide a risk estimate such as a hazards ratio or risk ratio;
  - **No**, if the study information indicates that a study drug is NOT compared to an active or inactive comparator as above;
  - **Unknown**, if it is not clear whether there is a formal comparative evaluation or not.
- **Setting**, using information from **the protocol** and/or **section 10 and 13** of the EU-PAS register**:**
  - **Routine**, if the data used come from routine clinical practice, i.e. without a study-specific treatment protocol;
  - **Experimental**, if the data used concern drug treatment based on a study-specific protocol;
  - **Unknown**, if the setting is not apparent;
  - **Not applicable**, if the study is a survey or review or meta-analysis.
- **Scope_Disease epidemiology,** based on **section 11** of the EU-PAS register form:
  - **Yes;**
  - **No.**
- **Scope_Risk_assessment,** based on **section 11** of the EU-PAS register form:
  - **Yes;**
  - **No.**
- **Scope_Drug utilisation study,** based on **section 11** of the EU-PAS register form:
  - **Yes;**
  - **No.**

- **Scope_effectiveness_evaluation,** based on **section 11** of the EU-PAS register form:
  - **Yes;**
  - **No.**
- **Scope_other,** based on **section 11** of the EU-PAS register form:
  - **Yes;**
  - **No.**
- **Specific_other_scope,** based on **section 11** of the EU-PAS register form:
  - **Insert free-text as reported in section 11.**
- **Paediatric_population_<18_years**, if from **section 8** of the EU-PAS register form, the study aims to include a paediatric (even if not exclusively so) population. This field is already compiled through a linkage with other studies in the EU-PAS register, but please check that they are correct:
  - **Yes;**
  - **No.**
- **Adult_population_18-65_years**, from **section 8** of the EU-PAS register form:
  - **Yes;**
  - **No.**
- **Elderly_population_>65_years**, from **section 8** of the EU-PAS register form:
  - **Yes;**
  - **No.**
- **Population_age_unknown**, from **section 8** of the EU-PAS register form:
  - **Yes;**
  - **No.**
- **Special_population_pregnant_women**, from **section 8** of the EU-PAS register form:
  - **Yes;**
  - **No.**
- **Special_population_lactating_mothers**, from **section 8** of the EU-PAS register form:
  - **Yes;**
  - **No.**
- **Special_population_renal_impairment**, from **section 8** of the EU-PAS register form:
  - **Yes;**
  - **No.**
- **Special_population_hepatic_impairment**, from **section 8** of the EU-PAS register form:
  - **Yes;**
  - **No.**
- **Special_population_immunocompromised**, from **section 8** of the EU-PAS register form:
  - **Yes;**
  - **No.**
- **Special_population_Other**, from **section 8** of the EU-PAS register form:
  - **Yes;**
  - **No.**
- **Specific_other_special_population_description**, from **section 8** of the EU-PAS register form:
  - **Insert free-text as reported in section 8.**
- **Drug_type**, i.e. one of the following based on the **protocol** and/or **sections 1, 6 and 12** of the EU-PAS Register, only for the main study drug and not for comparators:
  - **Non-biologic;**
  - **Biologic;**
  - **Biologic and non-biologic;**
  - **None;**
  - **Unknown**, if this information is not available from the web site or in the attached documents.

**Orphan_drug status**, based on the **protocol** and/or **sections 1, 6, and 12** of the EU-PAS Register, only for the main study drug and not for comparators. To assign orphan drug status or otherwise:

1. Look up the drug status on Orphanet by searching the <drug name> AND <Orphanet> in your browser. There seems to be a problem when looking up medicines directly on the Orphanet website but this is resolved if the search is done directly in an internet browser (e.g. Google).

2. If, according to Orphanet, the drug has an orphan designation in any country for the same indication of use as the disease under study in the EU-PAS register, consider the drug an orphan drug.

The options are as follows:

- - **Yes;**
  - **No;**
  - **Unknown.**
- **Publications_available**: Publications in PubMed in English or at least with the abstract or title in English. This information may be in the EU-PAS Register form **section 18** and/or **19**. If not, the publication should be searched on PubMed and Google Scholar, using the study’s official title, keywords or the acronym or the lead investigator surname.
  - **Yes;**
  - **No.**
- **Publication_DOI_or_URL**: This is information which should be reported for the publications identified.
  - **Free-text**, copy and paste the publication DOI or URL.
- **Difficulty_with_specific_item**: From the drop-down menu, select which specific item/s you were reasonably unsure of, based on the names of the variables
- **Description_of_difficulty**: Describe your specific difficulties
  - **Free-text.**
