# Supplementary figures and images for: Overview of the European post‐authorisation study register post‐authorization studies performed in Europe from September 2010 to December 2018
Source: Pharmacoepidemiol Drug Saf. 2022 Feb 11;31(6):689–705. doi: 10.1002/pds.5413 (PMC9303697; doi:10.1002/pds.5413)

**Appendix Figure B1:** Cohen’s Kappa for key variables with 95% confidence intervals.


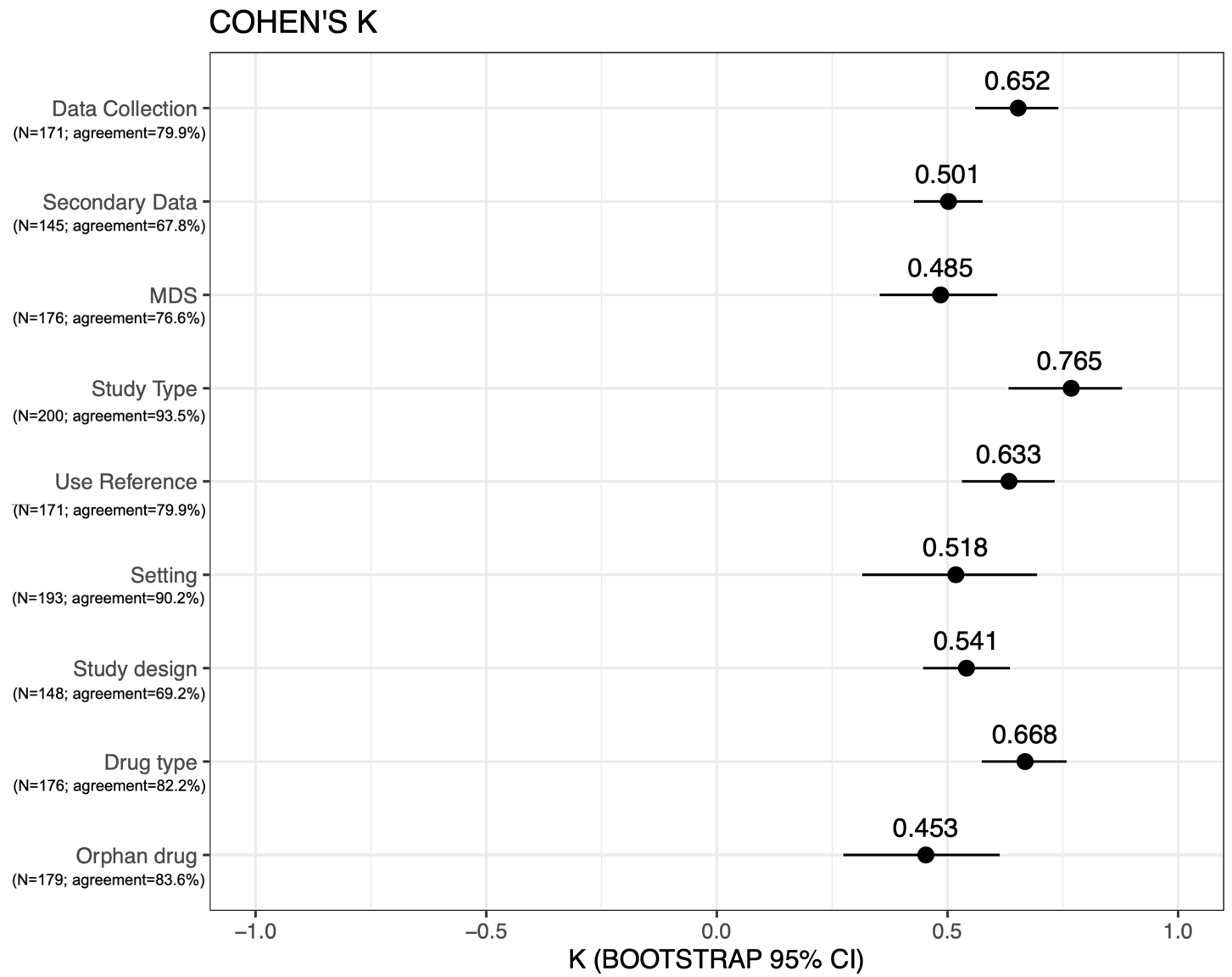

Supplement: Supplementary file 2 — Appendix Figure B1: Cohen's Kappa for key variables with 95% confidence intervals. [file PDS-31-689-s003.docx]
