# Supplementary material for: Overview of the European post‐authorisation study register post‐authorization studies performed in Europe from September 2010 to December 2018
Source: Pharmacoepidemiol Drug Saf. 2022 Feb 11;31(6):689–705. doi: 10.1002/pds.5413 (PMC9303697; doi:10.1002/pds.5413)
Supplement: Supplementary file 3 — Appendix Figure B2: Centre variations in Cohen's Kappa for key variables with 95% confidence intervals. [file PDS-31-689-s001.docx]

**Appendix Figure B2.** Centre variations in Cohen’s kappa for key variables with 95% confidence intervals


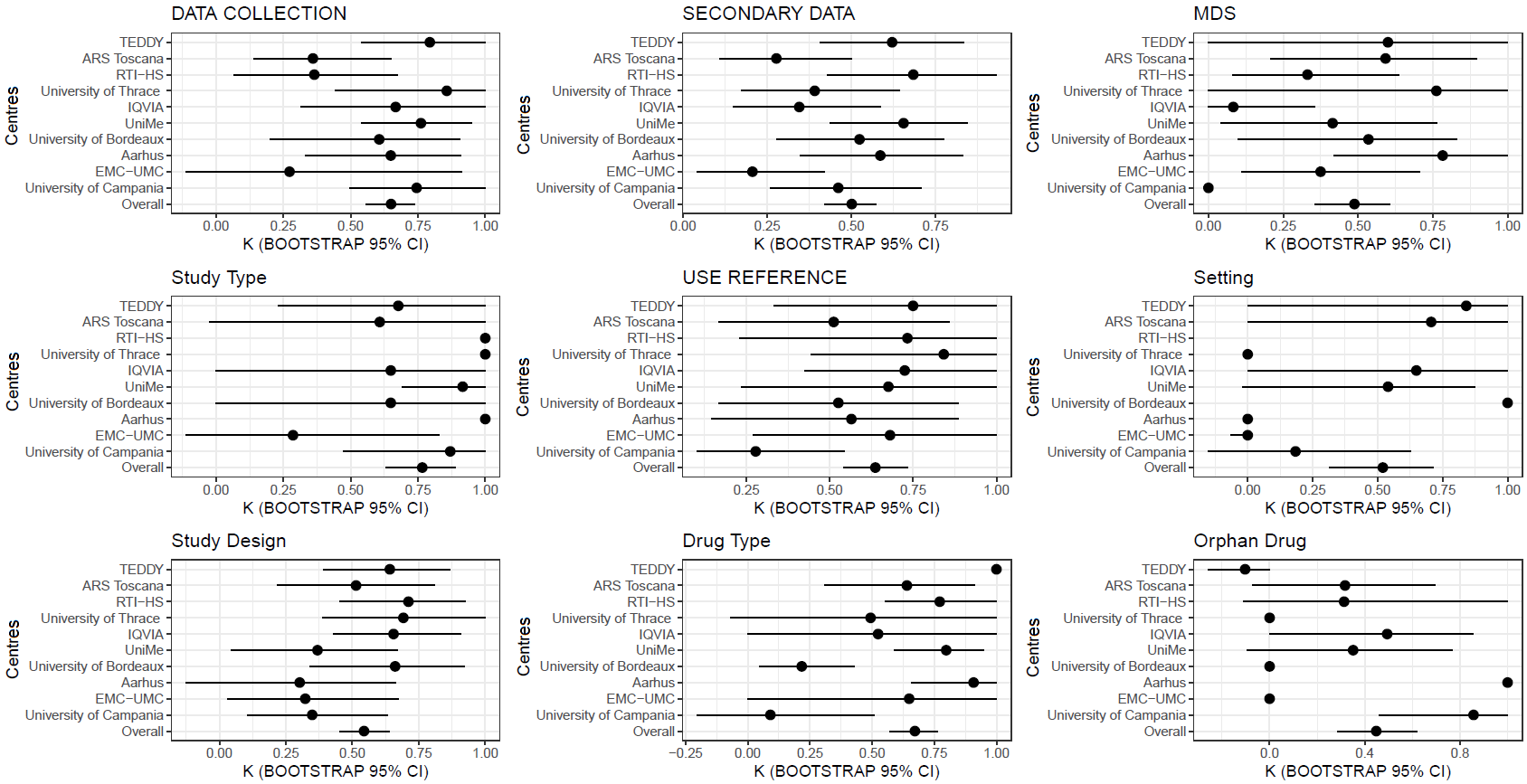


**Abbreviations:** MDS – multiple database study; CI – confidence interval
